# Precision Cardio-Oncology: Use of Mechanistic Pharmacokinetic and Pharmacodynamic Modeling to Predict Cardiotoxicities of Anti-Cancer Drugs

**DOI:** 10.3389/fonc.2021.814699

**Published:** 2022-01-10

**Authors:** Hai-ni Wen, Chen-yu Wang, Jin-meng Li, Zheng Jiao

**Affiliations:** ^1^ Department of Pharmacy, Shanghai Chest Hospital, Shanghai Jiao Tong University, Shanghai, China; ^2^ Department of Pharmacy, Affiliated Hangzhou Chest Hospital, Zhejiang University School of Medicine, Hangzhou, China

**Keywords:** cardiotoxicity, cardio-oncology, pharmacokinetic and pharmacodynamic modeling, mechanistic modeling, toxicity, anti-cancer drugs

## Abstract

The cardiotoxicity of anti-cancer drugs presents as a challenge to both clinicians and patients. Significant advances in cancer treatments have improved patient survival rates, but have also led to the chronic effects of anti-cancer therapies becoming more prominent. Additionally, it is difficult to clinically predict the occurrence of cardiovascular toxicities given that they can be transient or irreversible, with large between-subject variabilities. Further, cardiotoxicities present a range of different symptoms and pathophysiological mechanisms. These notwithstanding, mechanistic pharmacokinetic (PK) and pharmacodynamic (PD) modeling offers an important approach to predict cardiotoxicities and offering precise cardio-oncological care. Efforts have been made to integrate the structures of physiological and pharmacological networks into PK-PD modeling to the end of predicting cardiotoxicities based on clinical evaluation as well as individual variabilities, such as protein expression, and physiological changes under different disease states. Thus, this review aims to report recent progress in the use of PK-PD modeling to predict cardiovascular toxicities, as well as its application in anti-cancer therapies.

## Introduction

Advances in cancer treatment have dramatically improved patient survival rates. At the same time, however, the issue of preventing and managing treatment-associated chronic adverse events has become increasingly important. Cardiovascular complications have been identified as one of the leading causes of mortality in cancer survivors, regardless of the cancer type (1, 2). This has led to the development of a novel field, cardio-oncology, which focuses on reducing or managing the cardiotoxicity of anti-cancer agents, while maximizing therapeutic effects and managing patients with cancer having cardiovascular comorbidities. Further, cardio-oncology is increasingly becoming part of the standardized care for patients with cancer (3), and cardiovascular complications associated with cancer therapies, including arrhythmia, hypertension, and heart failure, have been observed in clinical practice. Furthermore, the mechanisms behind these clinical symptoms can be categorized into: (1) drug-induced electrocardiograph changes; (2) drug-induced hemodynamic changes; and (3) drug-induced changes in molecular signaling pathways (4).

Pharmacokinetic and pharmacodynamic (PK-PD) modeling is an approach by which concentration-driven drug effects can be quantitatively predicted. Traditionally, in PK models, multiple compartments are applied to describe the kinetic behaviors of therapeutic drugs, with the different compartments representing various organs or tissue levels, within which the action of the relevant drugs is kinetically consistent. Additionally, in classical PD models, empirical mathematical models are used to describe drug effects. Therefore, by offering the possibility to gain a deeper understanding regarding basic pharmacology and with the development of computational capacities, mechanistic PK-PD modeling can be used for the integration of physiological and pharmacological mechanisms (5).

Further, mechanistic PK-PD modeling is an emerging field, the definition of which is constantly evolving (6). Specifically, basic mechanism-based PK-PD modeling often incorporates one or more critical drug action steps, such as receptor binding or cell turnover, to capture major rate-limiting steps in drug dispositions and explain between-subject variabilities (7). Furthermore, systems pharmacology modeling provides a comprehensive modeling approach that has as objective to integrate the structures of physiological and pharmacological networks through PK-PD modeling (8, 9). On the one hand, the physiological-based pharmacokinetic (PBPK) modeling framework enables the mechanistic description of drug absorption, distribution, metabolism, and excretion processes at the physiological level. Thus, these mechanistic descriptions can be extrapolated to different populations and disease states if the associated physiological changes can be elucidated (10). On the other hand, with the development of high-throughput analytical methods, bioinformatics, and system biology, quantitative systems pharmacology (QSP) aims to quantitatively describe the behaviors of biological systems, and explain between-subject variabilities at genetic, protein, cellular, and whole-body levels (9).

To date, mechanistic PK-PD modeling has been extensively applied to quantify the cardiovascular toxicities of therapeutic drugs and predict the toxicities of anti-cancer drugs. In a few studies, mechanistic models have been established to describe the cardiotoxicities of anti-cancer drugs. Therefore, in this work, our aim was to review the role of mechanistic PK-PD modeling with respect to cardiovascular safety and its application in cardio-oncology.

## Modeling of Changes in Electrocardiograph

### Mechanisms of Drug-Induced Changes in Electrocardiographs

Several patients with cancer experience arrhythmias that are associated with anti-cancer therapies or cancers (11). Further, drug-induced arrhythmias can lead to life-threatening adverse events or sudden death, and clinically, this is frequently evidenced by changes in the electrocardiographs of patients (12). Among the various forms of arrhythmias, torsades de pointes (TdP) are the most dangerous. Specifically, TdP, meaning “twist of the points”, is a polymorphic ventricular tachycardia (VT) that is potentially fatal, and given that its occurrence is associated with QT interval prolongation, QT intervals are widely recognized as a proxy for TdP, as well as an index of cardiovascular safety (13). Thus, the quantitative modeling of QT intervals is the most popular strategy by which the proarrhythmic properties of a given drug can be clarified.

### Basic Mechanism-Based Modeling of Electrocardiograph Changes

The drug concentration-driven prolongation of QT intervals can be quantitatively predicted using PK-PD modeling. Additionally, the response of the QT interval to anti-cancer drugs has been successfully described empirically for several drugs, including dofetilide, azithromycin, and moxifloxacin, using (log-) linear models as well as simple and sigmoid *E*_max_ models (4). As the QT interval is strongly dependent on factors, such as heart rate and circadian rhythm, several attempts have been made to model it by correcting for these factors. For example, Chain et al. established a PD model to describe the corrected QT interval as a function of both physiological conditions and drug effects (14), as expressed below.


$$QT_{C}=QT_{0}\times RR^{\alpha }+A\times \cos\left(\frac{2\pi }{24}\left(t-\phi \right)\right)+Slope\times C$$

where QT_0_ represents the baseline for the QT-RR relationship, RR represents the interval between the R waves on the electrocardiogram, α represents an individual correction factor, the cosine function describes the circadian rhythm of the heart in different phases, and *C* represents drug concentration. In this case, the drug effect was modeled linearly.

Additionally, the QT interval is a sensitive but non-specific index of cardiac safety. In fact, several drugs share the same QT interval prolongation effect, but have different proarrhythmic properties (15). Thus, another biomarker of drug-induced arrhythmic risk, the human Ether-à-go-go-related Gene (hERG) channel block (16), has been identified. Arrhythmic risk is presumed to be dependent on the affinity of the drug in question to the different ion channels that control the action potential (AP) duration of the heart. Therefore, the half-maximal inhibitory concentration (hERG IC_50_) value of a compound, which is defined as the concentration of a given drug that will decrease the current flow through the hERG channel by 50%, can be used to indicate the potency of a given drug to induce TdP. With the aid of mathematical cardiac electrophysiology models, drug-ion channel interactions have been mechanistically modeled to predict the effects of drugs on AP duration. For example, Mirams et al. (17) predicted the TdP risks associated with various drugs using their reported hERG IC_50_ values. Specifically, the conductance of a given channel, *j* (g_j_), as a function of the drug amount (D) and the IC_50_ value can be modeled as follows:


$${\text{g}}_{j}=g_{control,j}{\left(1+{\left(\frac{\left(D\right)}{\left(IC_{50}\right)j}\right)}^{n}\right)}^{-1}$$

where g_control, j_ represents the baseline maximal conductance of channel *j*. Additionally, the conductance of the channel can then be linked to channel currents and membrane voltages to predict changes in AP duration. In this regard, the application of cardiac electrophysiology models has enabled the classification of compounds as high-, intermediate-, and low-risk compounds with respect to the occurrence of TdP.

### Systems Pharmacology Modeling of Electrocardiograph Changes

**Figure 1:**
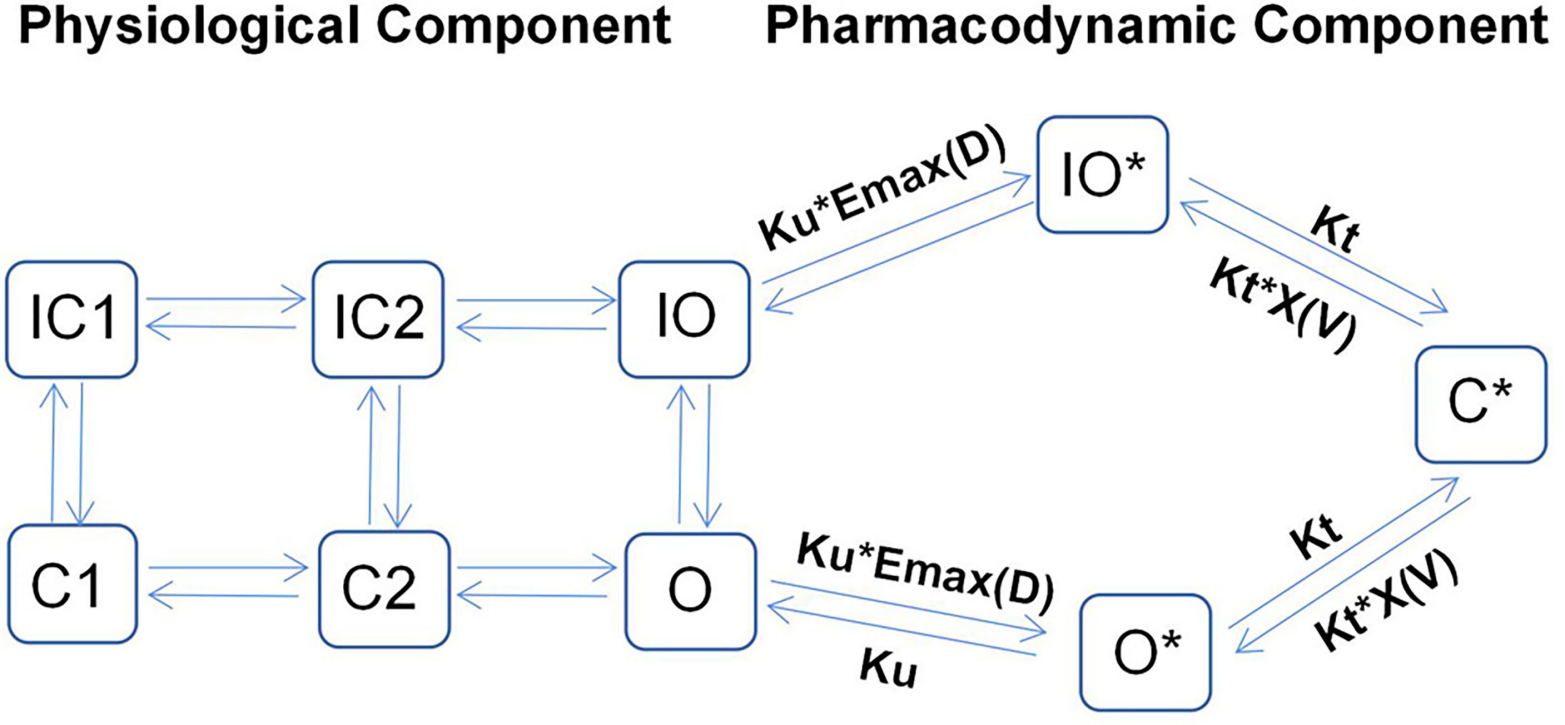
Illustrative structure of a dynamic hERG-binding model. The left part corresponds to the physiological component of the model, where C1 and C2 represent the closed states of the channel and O represents the open state, with the corresponding inactivated states indicated as IC1, IC2, and IO, respectively. The right part represents the pharmacodynamic component, which assumes three drug-bound states: open bound (O*), inactivated open bound (IO*), and closed bound (C*). The drug can be trapped in the C* state.

Recent studies have shown that cardiac electrophysiology models fail to capture the binding dynamics in drug-channel interactions. Thus, they cannot be used to distinguish between drugs with different binding rates to ion channels. In this regard, to further improve the prediction of drug-induced arrhythmic risks, Li et al. (18) proposed a novel hERG model that integrates cardiac electrophysiology and multi-ion channel pharmacology, as illustrated in **Figure 1**.

By applying a PD model with three drug-bound states, the model proposed by Li et al. can be used to distinguish the proarrhythmic risks associated with trapped compounds from those associated with their untrapped counterparts, as the former often have higher proarrhythmic risks for the same hERG IC_50_ value. The left-hand side of **Figure 1** shows the physiological part of the model, which describes the closing (C), inactivated closing (IC), and opening (O) states of ion channels. Conversely, the pharmacodynamic part (right-hand side) assumes three drug-bound states: open bound (O*), inactivated open bound (IO*), and closed bound (C*). This implies that the drug in question can be trapped in the C* state, implying that this proposed model can be used to successfully predict the TdP risk levels of all training compounds (n = 12).

Even though system pharmacology modeling can be used to describe the binding dynamics of drugs, parameters such as E_max_ and IC_50_ can only be estimated based on preclinical studies. Thus, the model needs measurable patient parameters patients such as the QT interval before its use can be extended to clinical practice.

## Modeling of Hemodynamic Changes

### Mechanisms of Drug-Induced Hemodynamic Changes

Blood pressure (BP) elevations and heart failure, which are common cardiovascular side effects of anti-cancer drugs, are often associated with hemodynamic changes. Specifically, hemodynamics is the study of blood flow dynamics, which are governed by BP and vascular resistance in different parts of the system, as well as by the contractability of the heart. Unlike TdP, BP elevations are not typically life-threatening, thus they have received less attention from pharmacometricians. Conversely, the occurrence of heart failure, which involves both hemodynamic and pathological changes, can be chronic and acute. Additionally, heart failure could also be the consequence of the direct cardio-toxicity of anticancer drugs, such as trastuzumab and anthracyclines (19, 20). Therefore, the modeling of heart failure is complicated and specific to a certain class of drugs.

### Basic Mechanism-Based Modeling of Hemodynamic Changes

Empirical PD models are frequently used to describe drug-induced BP elevations. For example, a linear function with a cyclical diurnal variation of mean aortic BP (MBP) has been applied in a PD model of regorafenib (21).


$$\begin{array}{c} BP=E_{0}+slope\times C\\ E_{0}=E_{BL}+A\text{mp}\times \cos\left(\frac{2\pi \left(t-Tshift\right)}{Freq}\right) \end{array}$$

where E_0_ represents baseline BP, which is influenced by the circadian rhythm. Further, C represents drug concentration, which is linked to response *via* a linear function.

**Figure 2:**
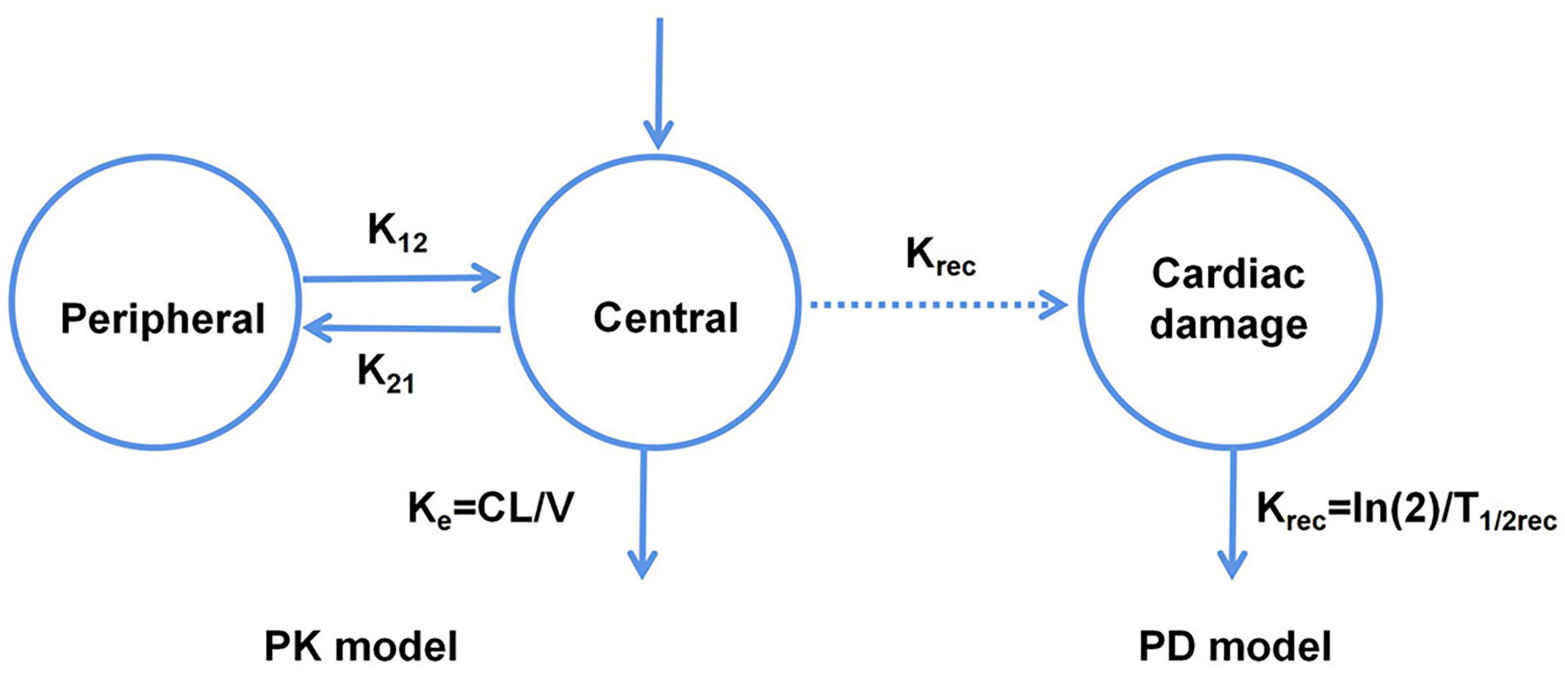
Schematic representation of the PK-PD model corresponding to the relationship between the left ventricular ejection fraction and trastuzumab exposure.

Van Hasselt et al. (22) developed a population PK-PD model corresponding to the relationship between the left ventricular ejection fraction (LVEF) and trastuzumab exposure. They also identified the associated clinically relevant covariates (**Figure 2**), and observed that the LVEF values could be best described using an effect-compartment model. Additionally, the population’s LVEF recovery half-life after trastuzumab treatment (T_1/2rec_) was estimated to be 49.7 d, and the cumulative anthracycline dose was found to be a significant determinant of the half-maximal effect concentration (EC_50_). Further, they also observed that anthracycline caused a 45.9% increase in sensitivity (i.e., a decrease in EC_50_) at its maximum cumulative dose.

### Systems Pharmacology Modeling of Hemodynamic Changes

**Figure 3:**
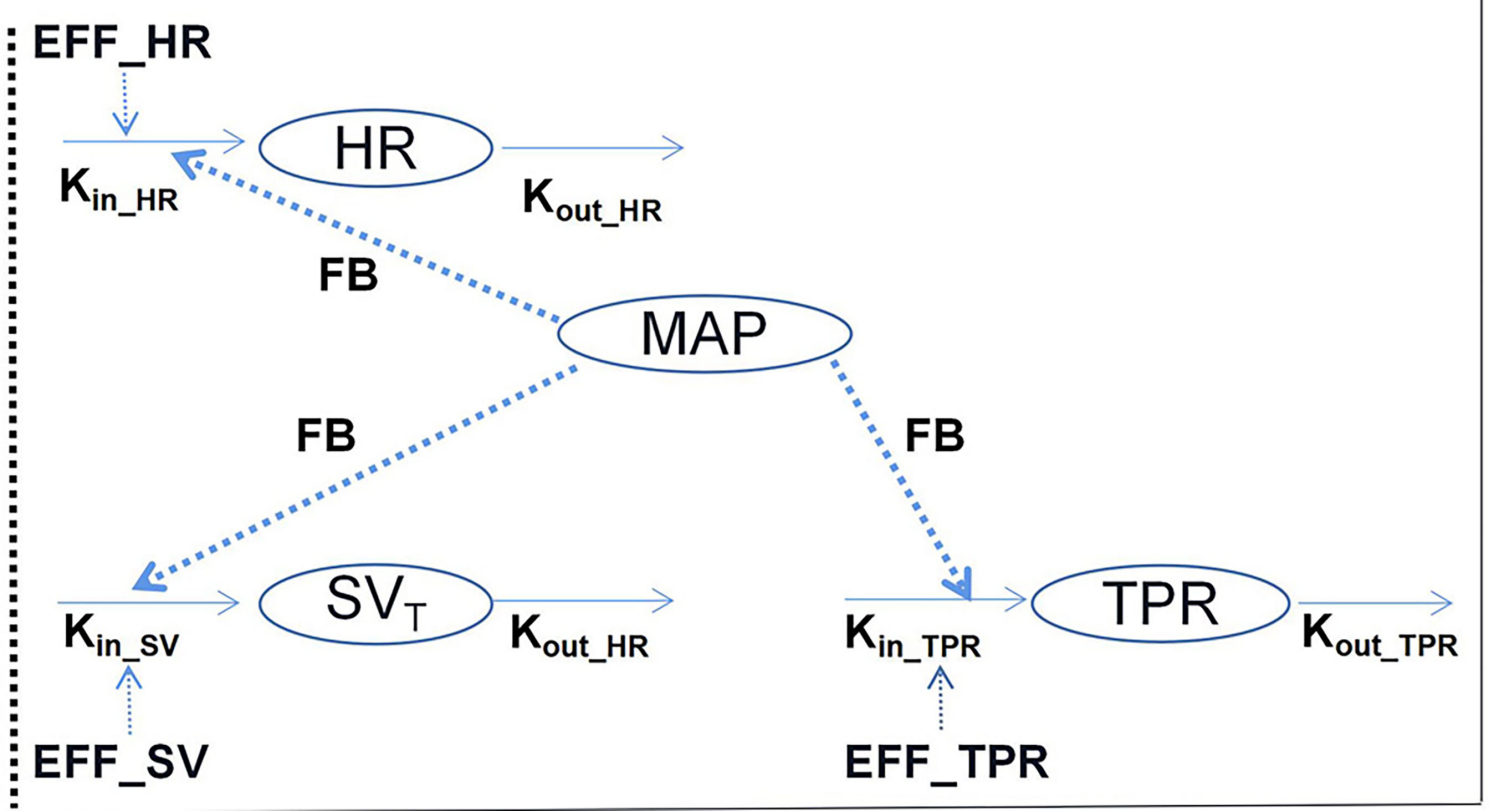
Illustrative structure of the hemodynamic system pharmacology model developed by Snelder et al. (23). HR, heart rate; TPR, total peripheral resistance; MAP, mean arterial pressure; and SV, stroke volume. FB- represents the negative feedback mechanism through MAP. The effects on HR, SV, and TPR are described using turnover models. K_in_HR_, K_in_SV_, and K_in_TPR_ represent the zero-order production rate constants and k_out_HR_, k_out_SV_, and k_out_TPR_ represent the first-order elimination rate constants.

Traditionally, hemodynamic parameters, such as BP or heart rate (HR), are often quantified independently, without considering the inter-relationships between them. Important variables for cardiovascular hemodynamics include: HR; mean arterial, diastolic, and systolic BP (MAP, DBP, and SBP, respectively); stroke volume (SV); cardiac output (CO); and total peripheral resistance (TPR), and the interrelationships between MAP, TPR, CO, HR, and SV are expressed as: (i) MAP = CO × TPR and (ii) CO = HR × SV (4). Further, the interrelationships between these variables are complex owing to the feedback mechanism of hemodynamics. Therefore, to compute these variables simultaneously, a systems approach that integrates cardiovascular physiology and the interactions between these variables is needed. In this regard, Snelder et al. (23) proposed a systems model with negative homeostatic feedback through MAP that can be used to describe changes in TPR, HR, and SV, as illustrated in **Figure 3**.

In the structure of this model, three turnover models that are regulated by homeostatic feedback through MAP (FB-MAP) are linked together to describe changes in HR, SV, and TPR. Additionally, in each equation, K_in__ *_i_* represents the zero-order production rate of each parameter, while k_out__ *_i_* represents the first-order elimination rate of each parameter.


$$\begin{array}{l} \frac{dHR}{dt}=K_{in\_HR}\times \left(1-FB\times MAP\right)-k_{out\_HR}\times HR\\ \frac{dSV^{*}}{dt}=K_{in\_SV}\times \left(1-FB\times MAP\right)-k_{out\_SV}\times SV^{*}\\ \frac{dTPR}{dt}=K_{in\_TPR}\times \left(1-FB\times MAP\right)-k_{out\_TPR}\times TPR\\ SV=SV^{*}\times \left(1-HR\_SV\times LN\left(HR/BSL\_HR\right)\right)\\ CO=HR\times SV\\ MAP=CO\times TPR \end{array}$$

Considering the circadian rhythm as well as drug effects, these equations can be written as follows:


$$\begin{array}{l} \frac{dHR}{dt}=K_{in\_HR}\times \left(1+CR_{HR}\right)\times \left(1-FB\times MAP\right)\times \left(1+EFF+HD_{HR}\right)-k_{out\_HR}\times HR\\ \frac{dSV^{T}}{dt}=K_{in\_SV}\times \left(1-FB\times MAP\right)\times \left(1+EFF\right)-k_{out\_SV}\times SV^{T}\\ \frac{dTPR}{dT}=K_{in\_TPR}\times \left(1+CR_{TPR}\right)\times \left(1-FB\times MAP\right)\times \left(1+EFF+HD_{TPR}\right)-k_{out\_TPR}\times TPR \end{array}$$

where CR*_i_* represents the circadian rhythm of each carrier and EFF represents drug effect, which for different drugs, is assessed based on linear, power, E_max_, or Sigmoid E_max_ models.

The abovementioned model has enabled the prediction of drug-induced hemodynamic changes based on HR and MAP measurements. More recently, Sang et al. (24) utilized the model for predicting anthracycline-induced heart failure, and by quantifying the interactions between preload, afterload, and the myocardial contraction of the cardiovascular system in the QSP model, they were able to distinguish pre-existing diseases or disease progression from drug effects. Further, in this study by Sang et al., the QSP-PK-PD model of doxorubicin-induced cardiotoxicity showed desirable prediction in a population consisting of individuals with and without preexisting cardiovascular conditions.

## Modeling of Drug-Induced Changes in Molecular Signaling Pathways

### Drug-Induced Changes in Molecular Signaling Pathways

While the effects of cancer drugs on electrocardiographs and the hemodynamic functions of the cardiovascular systems are a shared mechanism of drug-induced cardiotoxicities, in recent studies, more interest has been given to revealing the drug-specific mechanisms that underlie cardiotoxicities, especially with respect to impact on molecular signaling pathways (25).

The cardiac side effects of chemotherapies were first reported following the introduction of daunorubicin. Additionally, the cardiotoxicity of anthracyclines has been widely investigated since their introduction (26). Specifically, anthracycline-induced cardiomyopathy can occur at both early and late onset cancers, and the well-accepted mechanism of such cardiotoxicity involves the iron-dependent generation of reactive oxygen species (ROS), which thereafter cause oxidative damage to cardiomyocytes (27). Further, recent studies have revealed that ROS production is dependent on topoisomerase-2β, which seemingly, is a key mediator of doxycycline (DOX)-related cardiomyopathy (28).

In the past, the cardiac side effects of targeted therapies were initially considered minimal, as kinases were not constitutively active in normal tissues. However, the long-term use of targeted therapies still result in cardiovascular side effects, such as heart failure, QT interval prolongation, and myocardial injury. Further, considering tyrosine kinase inhibitors (TKIs) as examples, these treatments target the proliferation pathways of cardiomyocytes as well as cancer cells. Thus, the inhibition of these pro-survival kinases in normal cardiomyocytes results in the cardiotoxicities of TKI.

### Basic Mechanism-Based Modeling of Drug-Induced Changes in Molecular Signaling Pathways

It has been observed that anthracycline-induced cardiotoxicities are dose-dependent. Moreover, there seems to be a correlation between cardiotoxicity and drug peak plasma levels (29). Despite various proposed dosing strategies, such as the limiting of total dose and increasing infusion duration, the observed variability in individual responses to anthracyclines is still unclear. Therefore, PK-PD modeling provides a potential solution for anthracycline precision dosing.

**Figure 4:**
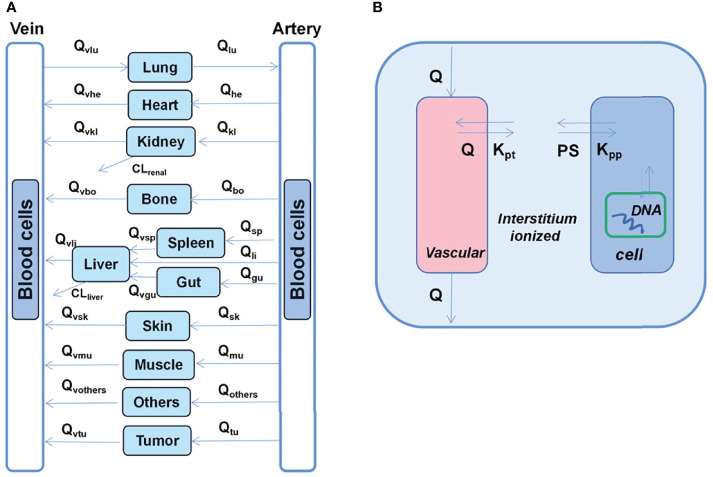
Schematic diagram of the developed multiscale PB-PK model for doxorubicin. **(A)** Whole-body PB-PK model comprising seven tissues and two blood compartments. The blood compartments are further divided into plasma and blood cell sub-compartments. **(B)** Tissue model; each tissue is divided into the vascular, interstitial, intracellular, and nucleus DNA-bound sub-compartments.

He et al. (30) developed a multiscale PK model that involves the assessment of doxorubicin dispositions as well as interstitial tissues, cells, and cellular organelles (**Figure 4**). Additionally, in most previous studies, it has been observed that cardiotoxicity is associated with the average plasma concentrations of different drugs. However, the most relevant concentrations with respect to cytotoxicity are those in cells or nuclei. In this regard, the nucleus sub-compartment equation was defined as follows:


$$\begin{array}{c} C_{\text{e}\_org}=0.5\times \left(\left(C_{et\_org}-CN_{org}-K_{d}\right)+\sqrt{{\left(C_{et\_org}-CN_{org}-K_{d}\right)}^{2}+4\times K_{d}\times C_{et\_org}}\right)\\ C_{DNA\_bound}=C_{et\_org}-C_{e\_org} \end{array}$$

where *C*_et_org_ represents total intracellular concentration, *C*_e_org_ represents free intracellular concentrations, *CN*_org_ represents DNA concentration, and *C*_DNA_bound_ represents DNA bound concentration.

The model predicted that prolonged infusion did not reduce doxorubicin-deoxyribonucleic acid (DNA) adducts at the tumor nucleus. This is consistent with clinical observations that prolonged infusion do not compromise the anti-tumor effect, indicating that DNA torsion is a primary anti-tumor mechanism (31).

### Systems Pharmacology Modeling of Drug-Induced Changes in Molecular Signaling Pathways

TKI-induced cardiotoxicity can be attributed to the activity of one or more tyrosine kinases in cardiomyocytes. Further, critical processes, such as survival signaling, energy homeostasis, and excitation-contraction coupling are controlled by molecular signaling pathways. Thus, QSP approaches seem to be well suited for the study of TKI-induced cardiotoxicity given that tyrosine kinase signaling encompasses large as well as complex networks with numerous feedback loops.

**Figure 5:**
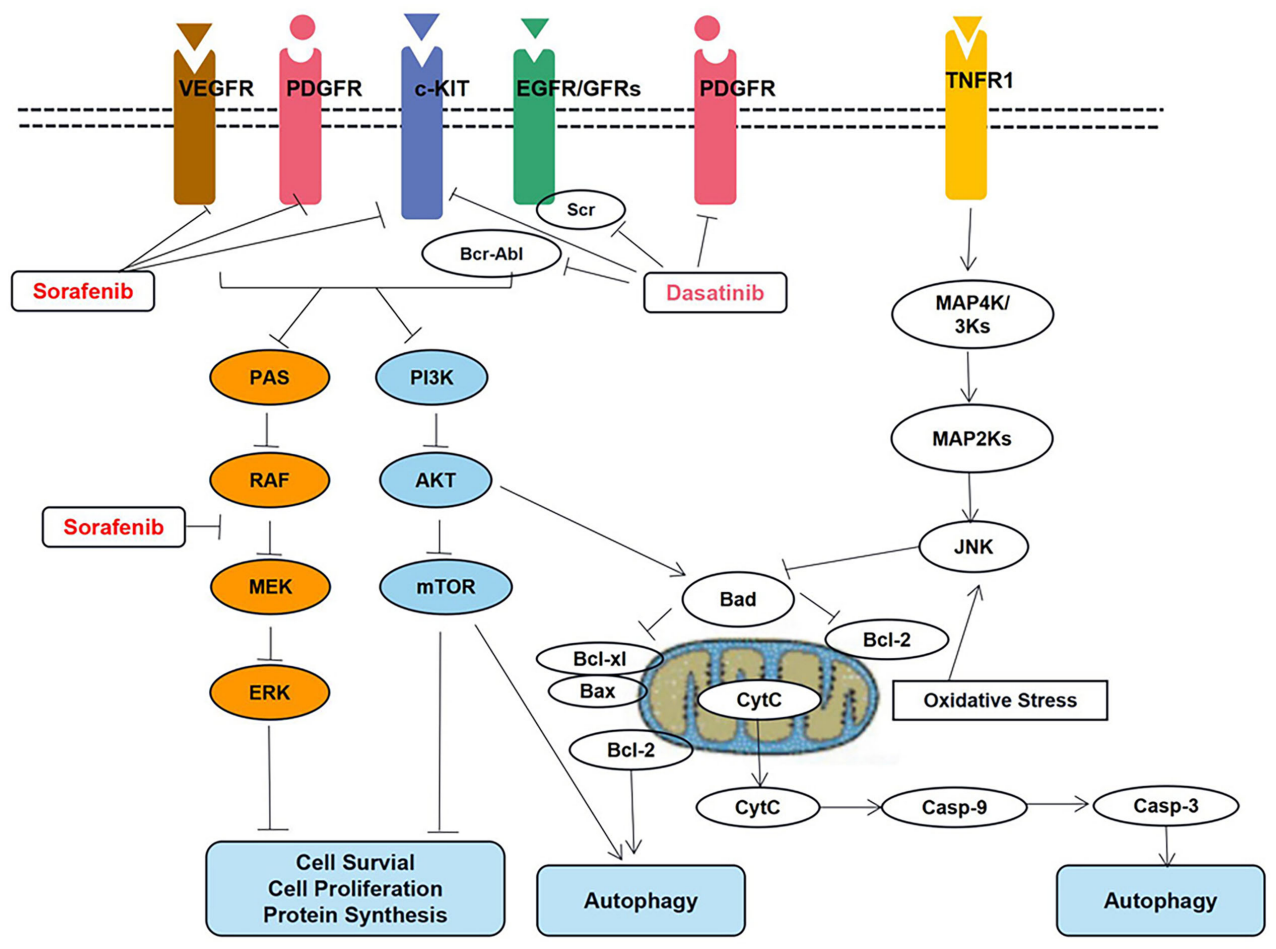
Key signal transduction pathways involved in dasatinib- and sorafenib-induced toxicity in cardiomyocytes. AKT, AKT8 virus oncogene cellular homolog; BAD, Bcl2-associated death promoter; BAX, Bcl2-associated X protein; Bcl-2, B-cell lymphoma 2 protein; Bcl-xl, B-cell lymphoma extra-large protein; Bcr-Abl, fusion protein encoded by the Philadelphia chromosome; Casp-3, caspase-3; Casp-9, caspase-9; c-KIT, stem cell growth factor receptor; Cyt, C cytochrome c; EGFR, epidermal growth factor receptor; ERK, extracellular signal regulated kinase; JNK, Jun N-terminal kinase; MAPK, mitogen activated protein kinase; MEK, MAPK/ERK kinase; mTOR, mammalian target of rapamycin; PDGFR, platelet derived growth factor receptor; PI3K, phosphoinositide 3 kinase; RAF, rapidly accelerated fibrosarcoma kinase protein; RAS, prototypical member of the Ras superfamily of proteins (belonging to the small GTPase group of proteins); Src Rous, sarcoma oncogene cellular homolog tyrosine kinase protein; TNFR1, tumor necrosis factor receptor 1; VEGFR, vascular endothelial growth factor receptor.

Vaidya et al. (32) recently investigated two TKIs, dasatinib and sorafenib. Further, QSP models have been developed to capture various trends in protein signaling and cellular responses regarding parameter estimates. In this regard, the key signal transduction pathways are shown in **Figure 5**.

The proteins in the apoptotic pathway that involves pBAD, pBcl2, Caspase-9, and active Caspase-3 have been described in the QSP model. Additionally, the model can be used to predict the IC_50_ values corresponding to different drug concentrations; these simulation results have been verified using data based on *in vitro* studies.

Additionally, the QSP platform is useful for elucidating cardiotoxicity mechanisms, and simulations based thereon can facilitate the evaluation of drug dosing strategies to the end of alleviating cardiotoxicity. Therefore, it offers the possibility to overcome the problem of cardiotoxicity without compromising the cytotoxic activity of the different drugs that are used to treat specific malignancies.

## Discussion

As an emerging field of interest, cardio-oncology aims to identify patients with risk factors, prevent cardiovascular damage, and monitor or manage the progress of cardiovascular toxicities (33). Mechanistic PK-PD modeling offers a potential approach for the prevention and identification of cardiovascular toxicities by quantifying exposure-response relationships. Limitations of PK-PD modeling should also be noted. First, while examples of mechanistic PK-PD modeling in cardiovascular safety with respect to anti-cancer drugs exist, they have been limited to a few drugs. Second, such PK/PD models should be further evaluated by large prospective clinical investigations before applying to the real clinical settings. Third, PK-Pd models could be considered as an additional tool to predict cardiac toxicity but they do not substitute to clinical evaluation. Complementary to clinical evaluations, further investigations of predictive performances are essential to their clinical applications.

Based on published studies, drug-induced electrocardiograph and hemodynamic changes can be sufficiently modeled using various model structures. Additionally, modeling techniques for electrocardiograph and hemodynamic changes are flexible and versatile, and pharmacometricians can choose the appropriate ones based on the purpose of modeling as well as the characteristics of the data used. However, these models lack information on the drug-specific mechanisms associated with cardiovascular toxicity, and their applications in clinical scenarios are limited. Therefore, in the future, drug-specific mechanisms can be incorporated into these models to enhance their performance with respect to predictabilities, and bridge the gaps between theoretical modeling and real clinical scenarios.

Additionally, the modeling of drug-induced changes in molecular signaling pathways tends to be comprehensive and drug-specific, and the prerequisite for successful modeling is an understanding of molecular pathways and dose-driven relationships. QSP provide the potential approaches given that they offer the possibility to construct biological interactions within systems. Moreover, QSP approaches can eventually be applied to distinguish disease populations from healthy ones, and also bridge the gap between application in ideal populations and real-world populations (34, 35). However, high-throughput experiments with system-level information, as well as computational techniques are required for the establishment of QSP networks. Thus, it is evident that QSP applications are still limited to preclinical research for some drugs of particular interest.

## Conclusions

In conclusion, mechanistic PK-PD modeling has been extensively applied to quantify the cardiovascular toxicities of anti-cancer drugs. Further, drug-induced changes in physio-electricity and hemodynamics can be well modeled using quantitative systems biology. Therefore, in future, bridging the gap between mechanistic cardiovascular models and clinical realities would offer the possibility to quantify the cardiovascular toxicities of anti-cancer drugs. Such PK/PD models should be further evaluated by large prospective clinical investigations before applying to the real clinical settings.

## Author Contributions

H-NW: Funding acquisition, visualization, writing — original draft, review, and editing. C-YW: Conceptualization, validation, writing — original draft, review, and editing. J-ML: Visualization, source, writing — review & editing. ZJ: Methodology, supervision, writing — original draft, writing — review, and editing. All authors contributed to the article and approved the submitted version.

## Funding

This study was supported by Shanghai “Rising Stars of Medical Talents” Youth Development Program-Youth Medical Talents: Clinical Pharmacist Program (SHWSRS(2021)_099), Bethune Charitable Foundation (B-19-H-20200622) and Wu Jieping Medical Foundation (320.6750.2020-10-103).

## Conflict of Interest

The authors declare that the research was conducted in the absence of any commercial or financial relationships that could be construed as a potential conflict of interest.

## Publisher’s Note

All claims expressed in this article are solely those of the authors and do not necessarily represent those of their affiliated organizations, or those of the publisher, the editors and the reviewers. Any product that may be evaluated in this article, or claim that may be made by its manufacturer, is not guaranteed or endorsed by the publisher.
